# Diagnostic accuracy of skin-prick testing for allergic rhinitis: a systematic review and meta-analysis

**DOI:** 10.1186/s13223-016-0126-0

**Published:** 2016-04-27

**Authors:** Immaculate F. Nevis, Karen Binkley, Conrad Kabali

**Affiliations:** Health Quality Ontario, 130 Bloor Street West, Toronto, ON M5S 1N5 Canada; Department of Medicine, University of Toronto, Toronto, ON Canada; Dalla Lana School of Public Health, University of Toronto, Toronto, ON Canada

**Keywords:** Skin-prick testing, Allergic rhinitis, Intradermal testing, Review, Meta-analysis, Diagnostic accuracy

## Abstract

**Background:**

Allergic rhinitis is the most common form of allergy worldwide. The accuracy of skin testing for allergic rhinitis is still debated. Our primary objective was to evaluate the diagnostic accuracy of skin-prick testing for allergic rhinitis using the nasal provocation as the reference standard. We also evaluated the diagnostic accuracy of intradermal testing as a secondary objective.

**Methods:**

We searched EBM Reviews from 2005 to March 2015; Embase from 1980 to March 2015; and Ovid MEDLINE(R) from 1946 to until March 2015. We included any study with at least 10 subjects including children. We excluded non-English studies. We performed data extraction and quality assessment using the QUADAS-2 tool.

**Results:**

We meta-analysed seven studies assessing the accuracy of skin-prick testing using the bivariate random-effects model, including a total of 430 patients. The pooled estimate for sensitivity and specificity for skin-prick testing was 85 and 77 % respectively. We did not pool results for intradermal testing due to few number of studies (n = 4), each with very small sample size. Of these, two evaluated the accuracy of intradermal testing in confirming skin-prick testing results, with sensitivity ranging from 27 to 50 % and specificity ranging from 60 to 100 %. The other two evaluated the accuracy of intradermal testing as a stand-alone test for diagnosing allergic rhinitis with sensitivity ranging from 60 to 79 % and specificity ranging from 68 to 69 %.

**Conclusions:**

Findings from this review suggest that skin-prick testing is accurate in discriminating subjects with or without allergic rhinitis.

## Background

Allergic rhinitis is a collection of symptoms that develop when the immune system becomes sensitized and overreacts to air-borne allergens [1]. It is the most common allergic disorder worldwide, [2] and one among the leading chronic conditions affecting both children and adults [3]. The global prevalence of allergic rhinitis is between 10 and 30 % for adults and as high as 40 % for children [4, 5]. Symptoms of allergic rhinitis usually develop before age 20 years, [6] and peak at age 20–40 years, before gradually declining [7].

The diagnosis of allergic rhinitis is often made on the basis of clinical characteristics and response to pharmacotherapy [7]. Evidence of sensitization to a known allergen usually involves a combination of skin or blood testing and patient’s exposure history [8]. Because of ease of administration and being less invasive, skin-prick testing is recommended for diagnosis of allergic rhinitis, followed by intradermal testing to confirm negative skin-prick testing results [9]. There is no universally accepted “gold standard” for detecting allergic rhinitis, although in research studies, nasal provocation is often used as the reference standard. There seems to be no consensus among researchers on the diagnostic accuracy of skin testing for allergies [10–12], including allergic rhinitis [13–15]. The variability in the accuracy of these tests across studies can be explained by lack of standardization, stability and composition of allergens, the testing device, the patient population, or the quality of study design. However, we are not aware of any systematic review that has evaluated the diagnostic accuracy of skin testing for allergic rhinitis across a range of studies. To address this issue, we conducted a systematic review and meta-analysis of published studies on the diagnostic accuracy of skin-prick testing in children or adults with suspected symptoms of allergic rhinitis. As a secondary analysis we also evaluated the diagnostic accuracy of intradermal testing for the same group of patients.

## Review

### Methods

We conducted and reported this review according to published guidelines using a pre-specified protocol [16].

### Eligibility criteria

We included any study that reported both sensitivity and specificity of skin-prick testing in at least 10 subjects including adults, children or both with allergic rhinitis using nasal provocation as the reference standard. We included full text papers and abstracts published in English language. We excluded studies enrolling subjects with known allergic status (commonly referred to as “case–control” designs in the diagnostic accuracy literature), and studies that did not include nasal provocation as the reference standard.

### Search strategy

We performed a literature search with the help of medical librarians on April 24, 2015, using All Ovid MEDLINE (from 1946 to present), Embase (from 1980 to present), Cochrane Database of Systematic Reviews (from 2005-present), Database of Abstracts of Reviews of Effects (from 1991-present), CRD Health Technology Assessment Database (from 2001-present), Cochrane Central Register of Controlled Trials (1991-present), and NHS Economic Evaluation Database (from 1995-present). The search strategy included a combination of key words and MeSH terms and was adapted for each database to account for differences in indexing. We limited our search to English language. We also searched gray literature sources and conference abstracts. Appendix 1 provides details on the search strategies used. We also examined reference lists for any additional relevant studies not identified through the search.

### Study selection, data abstraction and analysis

We screened titles and abstract (CK, IN) and obtained full texts for studies that met the eligibility criteria. We extracted estimates for sensitivity, specificity, and sample size from all eligible studies. We also computed sensitivity and/or specificity for studies that did not report these estimates but provided sufficient information for their derivation. We constructed forest plots to assess heterogeneity in test accuracy across studies. In case of substantial heterogeneity, we proceeded with a subgroup analysis to determine the reason for inconsistency. When homogeneity assumption was deemed appropriate, we pooled studies using the bivariate approach [17]. The pooled results were presented on a summary receiver operating characteristic curve (sROC), which included a 95 % confidence ellipse. When homogeneity assumption failed to hold, we presented sensitivity and specificity separately for each study. The logit transformation was used for the calculation of study specific confidence intervals to account for asymmetry in the distribution of sensitivity and specificity. When estimates were on, or too close to the boundary of the parameter space (i.e., values for sensitivity or specificity were equal to, or approximately equal to 0 or 100 %), a continuity correction factor of 1 % was applied. All analyses were performed using the MADA package in R version 3.0.2.

### Quality of evidence

The quality of evidence for each bivariate outcome within studies was examined according to the quality assessment of diagnostic accuracy studies (QUADAS-2) [18]. This tool consists of four key domains: patient selection, index test, reference standard, and flow and timing.

## Results

### Study selection

**Fig. 1:**
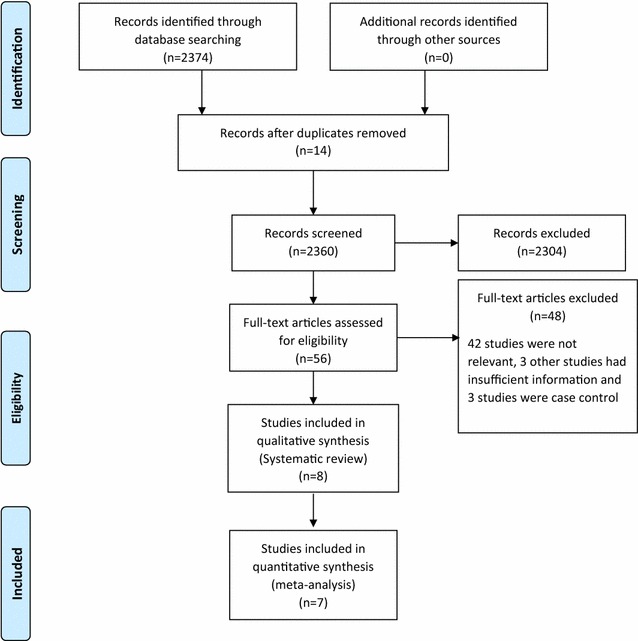
PRISMA flow diagram of studies identified, included and excluded

One reviewer (CK) screened and evaluated 2360 citations and assessed 56 full text articles for eligibility. An unbiased sample of 374 citations were screened by a second reviewer (IN) using the method of Nevis et al. [19]. The chance-corrected agreement for titles and abstracts was good (estimated kappa = 75 %; 95 % CI 50–100 %). We resolved disagreements by consensus. Of the 56 full text articles, we excluded 42 as they were not relevant, three articles had insufficient information on outcomes and three were case control studies. Figure 1 summarizes the selection process. Eight articles were eligible to be included in the systematic review [15, 20–25]. Only seven of the eight articles were included in the meta-analysis because one study restricted their allergen to *alternaria* that was not evaluated by any of the other eligible studies in this review, and whose findings deviated substantially from the remaining studies [14].

### Description of studies, methods and participants

Eight studies from four countries focused our primary research question (i.e., accuracy of skin prick testing), recruiting a total of 609 patients (range 37–141) (Table 1). Four of the included studies [14, 15, 20, 24] focused on secondary research question (i.e., accuracy of intradermal testing) (Table 2). Most studies were done in North America (*n* = 5), followed by one study each from Italy, Sweden and United Kingdom. All study participants were recruited using non-random sampling approaches. Five studies recruited participants in a clinical setting [15, 21–23, 25]. Most (n = 11) studies reported age of the study population, ranging from 9 to 70 years. The percentage of males ranged from 18 to 70 %. Seven of eight provided information on cut-off point for positive skin prick testing [20–25]. Five studies evaluated a single allergen, of which two evaluated cat allergens [24, 25] and the remaining three evaluated Timothy grass, ragweed and *alternaria* each [14, 15, 20]. Three studies evaluated two or more allergens [21–23] which included grass, mugwort, birch, pellitory, timothy, sweet vernal, cocksfoot, meadow fescue, rye, meadow and *dermatophagoides pteronyssinus* (Table 1). The most frequently evaluated allergen extract was timothy grass, reported in three studies [20, 22, 23] and cat, reported in two studies [24, 25].

**Table 1:** Characteristics of studies reporting primary outcome (skin prick testing)

Study, year	Country	Setting	Sample size	No. of males (%)	Mean age (range), years	Nasal provocation positive	Nasal provocation negative	Wheal size cut-off (mms)	Sensitivity, %	Specificity, %	Allergen extracts
Zarei et al. [25]	USA	Clinic	45	21 (47)	39 (25–66)	18	27	≥3^a^	100.0	74.1	Cat
Krouse et al. [14]	USA	Hospital	37	NR	NR (18–70)	15	22	≥3^a^	87.0	86.0	Timothy grass
Krouse et al. [14]	USA	Hospital	44	NR	NR (18–70)	19	25	≥3^a^	42.0	64.0	*Alternaria*
Gungor et al. [15]	USA	Unclear	62	NR	≥18	34	28	unclear	85.3	78.6	Ragweed
Wood et al. [24]	USA	Hospital	120	22 (18)	32 (18–65)	48	32	≥3^b^	79.0	91.0	Cat
Pastorello et al. [21]	Italy	Hospital	91	48 (53)	NR (9–57)	70	31	≥3 and 100,000BU/ml	98.0	70.0	Grass,mugwort, birch, pellitory, *Dermatophagoides pteronyssinus*
Petersson et al. [23]	Sweden	Hospital	69	48 (70)	27 (14–53)	36	33	≥0.5^c^	97.0	70.0	Birch and timothy grass
Pepys et al. [22]	UK	Hospital	141	NR	NR	72	64	≥1	68.1	84.4	Sweet vernal, cocksfoot, meadow fescue, rye, timothy, meadow

*NR* not reported

^a^Of the size of negative control

^b^Of the size of negative control plus 1.5 the size of positive control

^c^The size of positive control

**Table 2:** Characteristics of studies reporting secondary outcome (intradermal testing)

Study, year	Country	Setting	Sample size	Number of males	Age range, years	Nasal provocation positive	Nasal provocation negative	Wheal size cut-off (mms)	Sensitivity, %	Specificity, %	Allergen extracts
Krouse et al. [14]	USA	Hospital	37	NR	NR (18–70)	2	19	≥3^a^	50.0	100.0	Timothy grass
Krouse et al. [14]	USA	Hospital	44	NR	NR (18–70)	11	16	≥3^a^	27.0	69.0	*Alternaria*
Gungor et al. [15]	USA	Unclear	62	NR	≥18	34	28	≥2	79.4	67.9	Ragweed
Wood et al. [24]	USA	Hospital	120	22 (18)	32 (18–65)	10	29	≥6	60.0	68.9	Cat

*NR* not reported

^a^Of the size of negative control

### Primary analysis: diagnostic accuracy of skin-prick testing

**Fig. 2:**
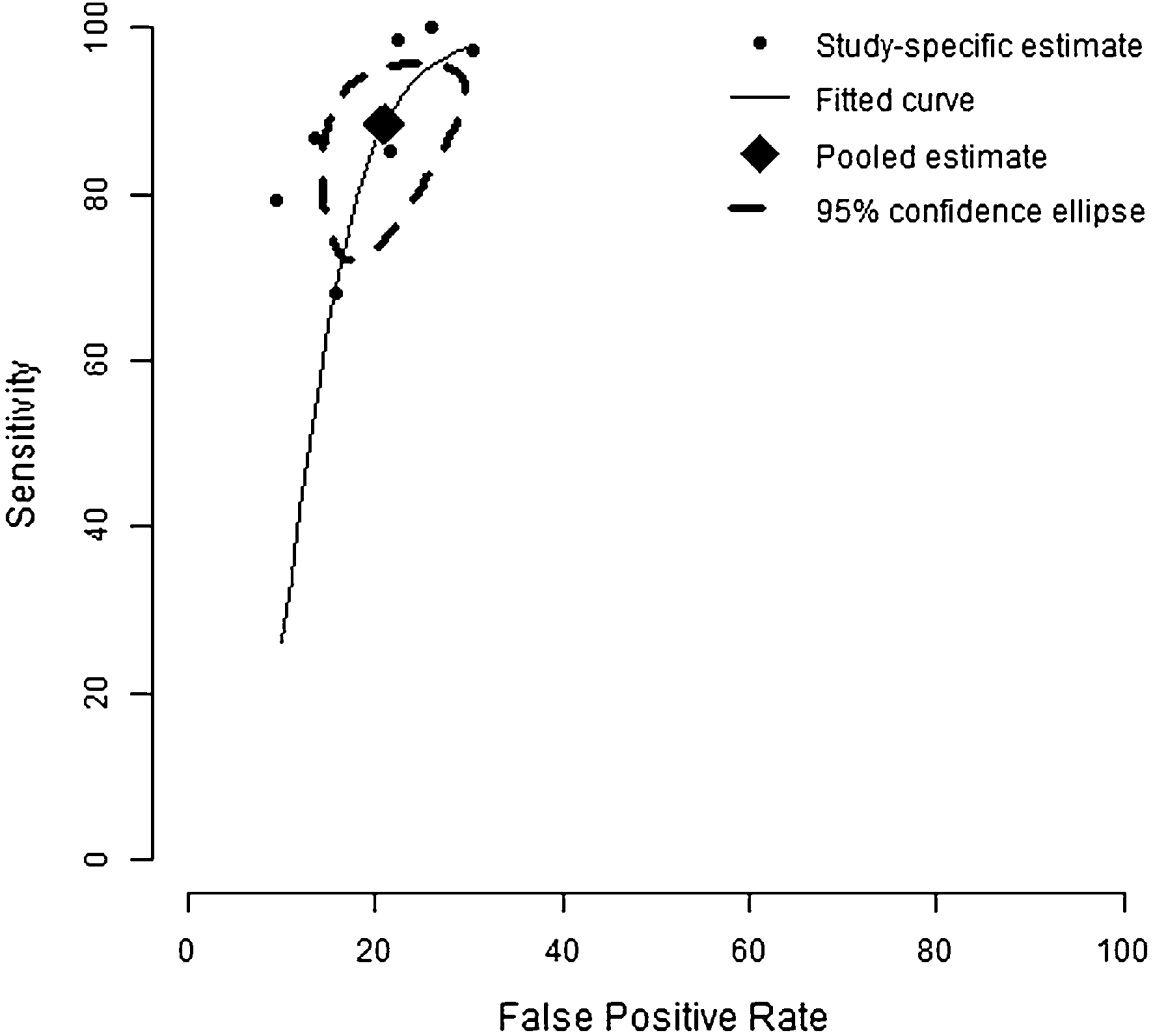
Summary receiver operating characteristic curve (sROC) of seven studies evaluating the accuracy of skin-testing for allergic rhinitis, plotted using a bivariate normal distribution model. Estimate of the pooled pair of sensitivity and specificity is 88.4 and 77.1 %

**Fig. 3:**
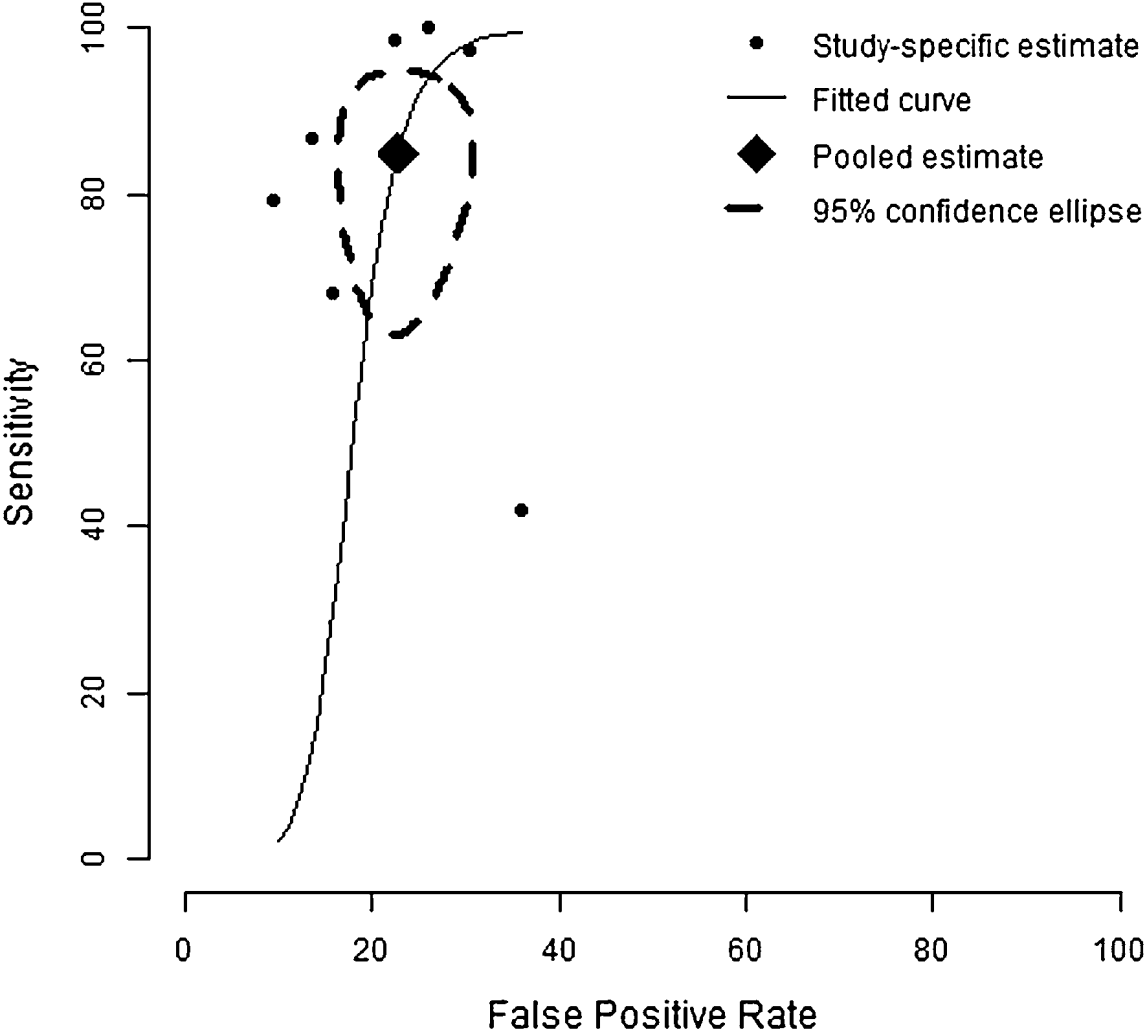
Summary receiver operating characteristic curve (sROC) showing the sensitivity of results for the accuracy of skin-testing for allergic rhinitis, when we include Krouse et al. [14]. Estimate of the pooled pair of sensitivity and specificity only fluctuates a little to 85.0 and 77.3 %

**Fig. 4:**
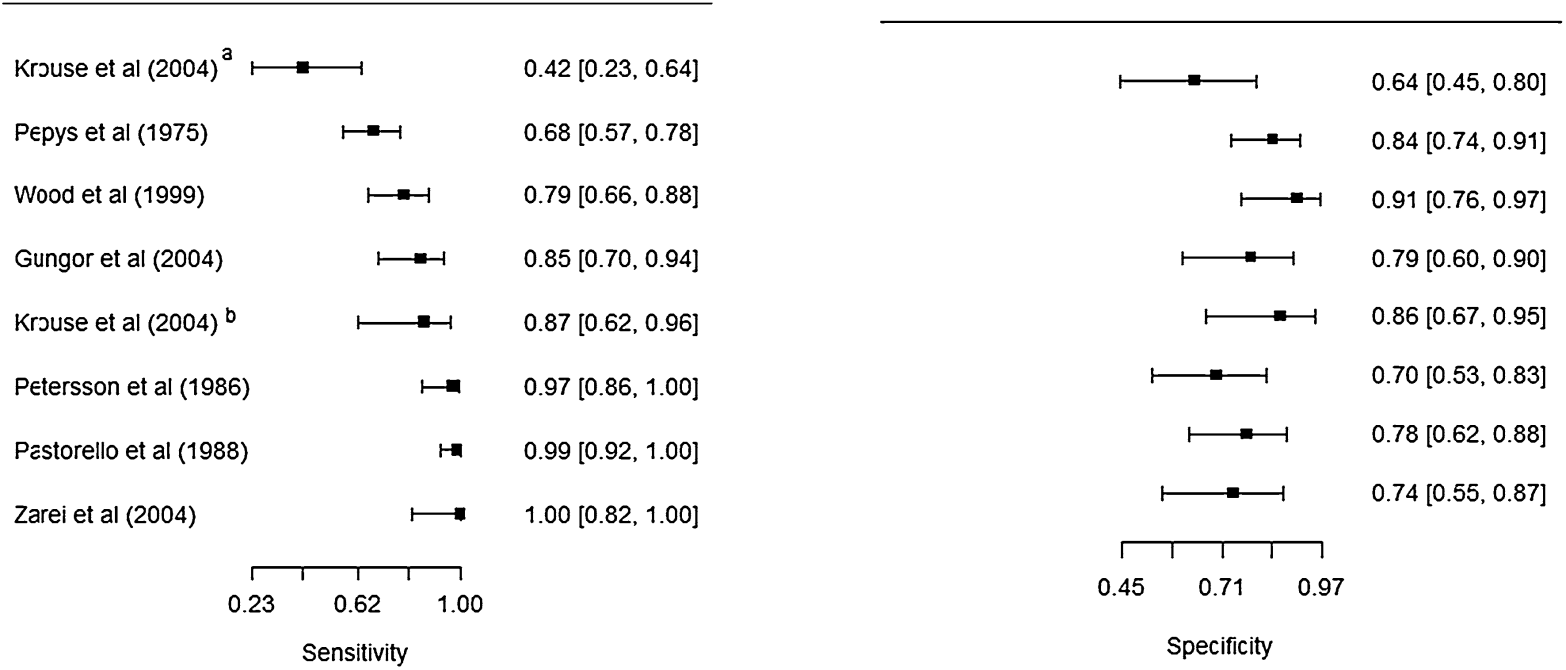
*Forest plots* for studies evaluating the accuracy of skin prick tests. Estimates from Krouse et al. [14]^a^ deviate considerably from the rest (its inclusion attenuates the negative correlation between sensitivity and specificity)

**Fig. 5:**
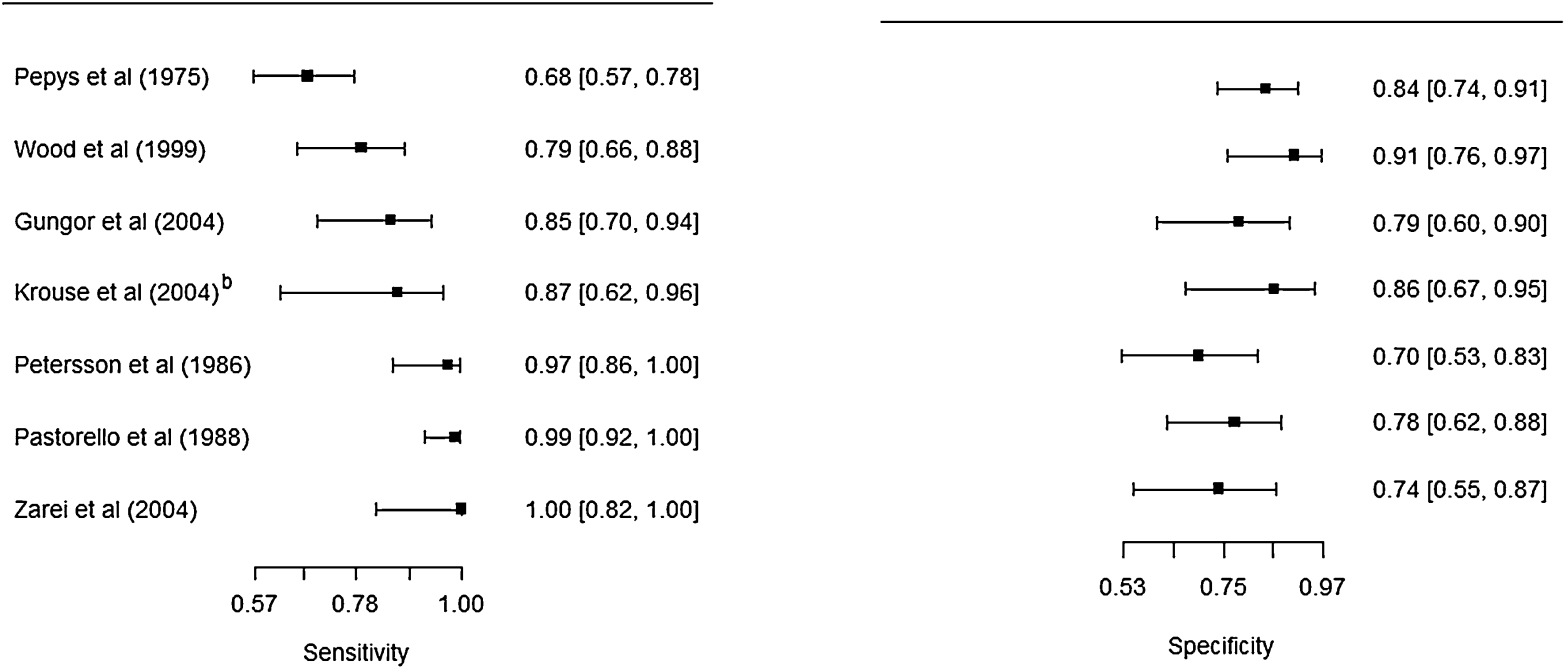
*Forest plots* for studies evaluating the accuracy of skin prick tests. Krouse et al. [14]^a^ is excluded

We conducted a meta-analysis of studies reporting sensitivity and specificity of skin-prick testing. The pooled estimate of sensitivity and specificity for this test was 88.4 and 77.1 % respectively (Fig. 2). We also conducted a sensitivity analysis by including in the meta-analysis, the study that tested for *alternaria* [14]. Inclusion of this study did not significantly alter the estimates for accuracy. The pooled estimate for sensitivity and specificity changed to 85.0 and 77.3 % respectively (Fig. 3). The forest plots for heterogeneity are presented in Figs. 4 and 5.

Five studies that evaluated the accuracy of skin-prick testing [14, 15, 20, 24, 25] restricted the analysis to single-allergen extracts. The sensitivity and specificity ranged from 79 % (95 % CI 66–88 %) to 100 % (82–100 %) and 79 % (95 % CI 66–88 %) to 91 % (76–97 %) respectively, excluding Krouse et al. [14]. When Krouse et al. [14] was included, the minimum values for sensitivity and specificity were altered to 42 % (95 % CI 23–64 %) and 64 % (95 % CI 45–80 %) respectively.

Three studies that evaluated the accuracy of skin-prick testing examined multiple-allergen extracts [21–23]. The reported sensitivity ranged from 68 % (57–78 %) to 97 % (86–100 %), and specificity ranged from 70 % (95 % CI 54–86 %) to 84 % (95 % CI 74–91 %) respectively.

### Secondary analysis: diagnostic accuracy of intradermal testing

We conducted a systematic review of four studies that reported sensitivity and specificity of intradermal testing. When intradermal testing was used to confirm negative skin-prick testing results, the estimates for sensitivity ranged from 27 % (95 % CI 10–57 %) to 50 % (sample size was too small for estimation of CI using asymptotic-based statistical tests) and those for specificity ranged from 69 % (95 % CI 51–83 %) to 100 % (95 % CI 83–100 %). When the test was evaluated as a stand-alone tool for diagnosing allergic rhinitis, the estimate for sensitivity was between 60 % (95 % CI 31–83 %) and 79 % (95 % CI 63–90 %), and that for specificity was 68 % (95 % CI 49–82 %). All four studies [14, 15, 20, 24] restricted the analysis to single-allergen extracts.

### Risk of bias and applicability concerns

**Fig. 6:**
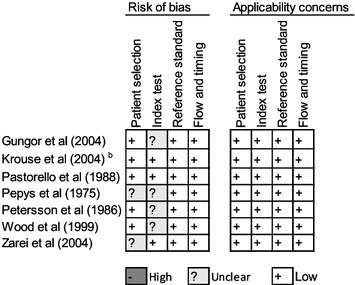
Reviewer’s judgment about the risk of bias in each included study that assessed the accuracy of skin-prick testing. See Appendix 2 for a detail explanation of domains for risk of bias and applicability concern

**Fig. 7:**
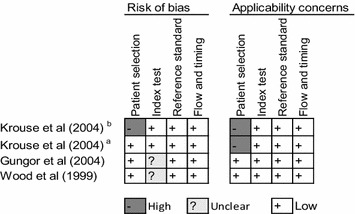
Reviewer’s judgment about the risk of bias in each included study that assessed the accuracy of intradermal testing. See Appendix 2 for a detail explanation of domains for risk of bias and applicability concern

**Fig. 8:**
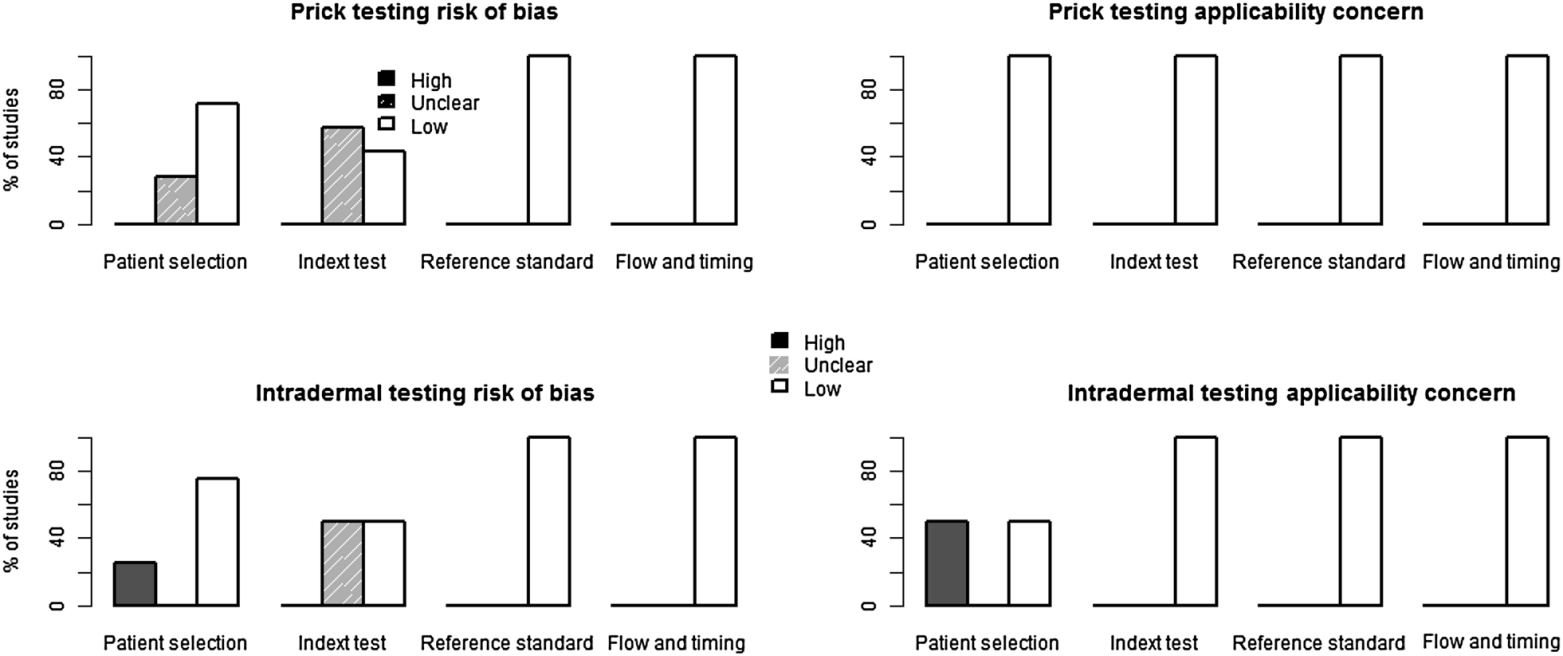
Methodological quality of the included studies. See Appendix 2 for a detail explanation of domains for risk of bias and applicability concern

We summarize assessment of risk of bias in Figs. 6, 7 and 8. For skin-prick testing the risk of bias was “unclear” in five studies [15, 22–25]. For intradermal testing the risk of bias was “high” in one study, [14] and “unknown” in two studies [15, 25]. Applicability concerns were “high” in two studies [14, 20].

We used Fig. 8 to evaluate the potential for heterogeneity in estimates for the accuracy of skin-prick testing. The inclusion of Krouse et al. [14] introduced a discernible heterogeneity across studies. Specifically, the 95 % confidence (CI) for sensitivity barely overlapped with CIs of other studies, and its inclusion swayed the correlation between sensitivity and specificity toward a positive value—violating a requirement for meta-analysis of diagnostic accuracy studies that the correlation should be non-positive for homogeneity assumption to hold. When this study was removed from the analysis, the negative correlation was observed (Fig. 5).

Five studies either did not report [15] or use [15, 22–24] a 3 mm cut-off value for the wheal size diameter recommended by the American Academy of Allergy, Asthma and Immunology (AAAAI) and the American College of Allergy, Asthma, and Immunology (ACAAI) [9]. Given the relation between the cut-off value and sensitivity and specificity, and because a 3 mm cut-off value might not be optimal in all settings, [26] we classified these studies as “unclear-risk of bias”. Moreover, the sample size for two studies evaluating the accuracy of intradermal testing [14, 20] was too small, calling into question whether findings from these studies apply to the majority of suspected allergic rhinitis patients presenting in clinics. We classified both studies as “high-risk of bias”.

## Discussion

Findings from this review suggest that skin-prick testing is reasonably accurate in identifying patients with suspected symptoms of allergic rhinitis. The level of accuracy reported in studies eligible for meta-analysis ranged from sensitivity of 68 to 100 % and specificity of 70 to 91 %. Although we could not establish the source of heterogeneity in testing accuracy across studies, several factors that influence accuracy of skin-prick testing have been reported in the literature [9, 27]. These include skill of the tester, the testing device, colour of the skin, skin reactivity on the day of testing, potency, and stability of test reagents.

To our knowledge this is the first systematic review and meta-analysis to evaluate the accuracy of skin-prick testing. Given lack of consensus among researchers and health practitioners on the performance of this test, findings from this review broaden our knowledge on the accuracy of this test across a large body of evidence. This is especially important given that effectiveness of intervention such as immunotherapy, avoidance, or pharmacotherapy largely depends on the correct diagnosis. Thus proper diagnosis can alleviate financial burden and loss in quality of life for millions of patients affected by allergic rhinitis.

Although there are no restrictions on age limits for skin-prick testing, literature suggests that skin reaction diminishes for young children [28]. That is, a 3 mm threshold for wheal size diameter is likely to yield a high rate of false positives in this group of patients. However, we were unable to assess the accuracy of skin-prick testing in children younger than 9 years due to the fact minimum age for eligible studies for this review was 9 years.

It should be noted that a 3 mm cutoff criteria recommended in guidelines is mainly based on reproducibility in relation to nasal provocation rather than clinical relevance [29]. That is, larger wheal sizes may predict a positive response to nasal provocation but not necessarily severity of clinical symptoms. The extent of agreement between wheal size and clinical symptoms may depend on population characteristics and allergen extracts.

We note the following limitations. First, we were unable to determine the degree of accuracy of intradermal testing because of the limitations in the four included studies. Hence, well designed methodologically rigorous studies are required to firmly establish the accuracy of intradermal testing. Second, we used nasal provocation as the reference standard. However, this test may not always represent the natural exposure to allergens. Despite this limitation, nasal provocation is still considered as the best “gold standard” available by several guidelines. Finally, there was a substantial variation in allergen extracts among studies. Nonetheless, skin-prick testing results remained fairly accurate regardless of the type of extracts.

## Conclusions

In conclusion this review supports findings from several studies that skin-prick testing is accurate for diagnosing patients with allergic rhinitis. Several factors have been reported to influence the accuracy of prick testing, including skill of the tester, the testing device, color of the skin, skin reactivity on the day of testing, potency, and stability of test reagents. We were unable to determine the degree of accuracy of intradermal testing because of the limitations in the four included studies. Well-designed methodologically rigorous studies are required to firmly establish the accuracy of allergy skin testing and especially intradermal testing.

## Appendix 1: Search strategy

Database: EBM Reviews—Cochrane Central Register of Controlled Trials <March 2015>, EBM Reviews—Cochrane Database of Systematic Reviews <2005 to March 2015>, EBM Reviews—Database of Abstracts of Reviews of Effects <1st Quarter 2015>, EBM Reviews—Health Technology Assessment <1st Quarter 2015>, EBM Reviews—NHS Economic Evaluation Database <1st Quarter 2015>, Embase <1980 to 2015 Week 16>, All Ovid MEDLINE(R) <1946 to Present>

## Appendix 2

See Table 3.

**Table 3:** Risks of bias and Applicability Judgements in Quadas-2

Domain	Patient selection	Index test	Reference standard	Flow and timing
Description	Describe methods of patient selection Describe included patients (previous testing, presentation, intended use of index test, and setting)	Describe the index test and how it was conducted and interpreted	Describe the reference standard and how it was conducted and interpreted	Describe any patients who did not receive standard or who were excluded from the 2 × 2 table (refer to flow diagram) Describe the interval and any interventions between index tests and the reference standard
Signalling questions (yes, no, or unclear)	Was a consecutive or random sample of patients enrolled? Was a case–control design avoided? Did the study avoid inappropriate exclusions?	Were the index test results interpreted without knowledge of the results of the reference standard? If a threshold was used, was it prespecified?	Is the reference standard likely to correctly classify the target condition? Were the reference standard results interpreted without knowledge of the results of the index test?	Was there an appropriate interval between index tests and reference standard? Did all patients receive a reference standard? Did all patients receive the same reference standard? Were all patients included in the analysis?
Risk of bias (high, low, or unclear)	Could the selection of patients have introduced bias?	Could the conduct or interpretation of the index test have introduced bias?	Could reference standard, its conduct, or its interpretation have introduced bias?	Could the patient flow have introduced bias?
Concerns about applicability (high, low, or unclear)	Are there concerns that included patients do not match the review question?	Are there concerns that the index test, its conduct, or its interpretation differ from the review question?	Are there concerns that the target condition as defined by the reference standard does not matched the review question?	

## Abbreviations

CRD Database: Centre for Reviews and Dissemination Database
NHS: National Health Service
MeSH: medical subject headings
sROC: summary receiver operating characteristic curve
QUADAS-2: quality assessment of diagnostic accuracy studies
95 % CI: 95 % confidence interval
ACAAI: American College of Allergy, Asthma, and Immunology
AAAAI: American Academy of Allergy, Asthma and Immunology
